# Cytokine-driven modulation of peripheral immune cell populations in Coronavirus disease 2019 patients

**DOI:** 10.3389/fimmu.2026.1733695

**Published:** 2026-04-07

**Authors:** Tanaka Arthur Choto, Bongiwe Ndlovu, Thajasvarie Naicker, Takafira Mduluza

**Affiliations:** 1 Optics and Imaging, Doris Duke Medical Research Institute, College of Health Sciences, University of KwaZulu-Natal, Durban, KwaZulu-Natal, South Africa; 2 HIV Pathogenesis Programme, Doris Duke Medical Research Institute, College of Health Sciences, University of KwaZulu-Natal, Durban, KwaZulu-Natal, South Africa; 3 Department of Biochemistry and Biotechnology, University of Zimbabwe, Harare, Zimbabwe

**Keywords:** COVID-19 hospitalised patients, COVID-19, IFN-γ, IL-10, IL-17A, leucocyte, lymphocyte, SARS-CoV-2

## Abstract

**Introduction:**

Coronavirus disease 2019 (COVID-19) is characterized by a spectrum of immune dysfunction, from mild illness to life-threatening hyper-inflammation. However, the specific relationships between individual cytokine expression profiles and peripheral immune cell distributions remain poorly defined, particularly in African populations.

**Methods:**

In this study, we investigated associations between tumour necrosis factor-α (TNF-α), interferon-γ (IFN-γ), interleukin-10 (IL-10), and interleukin-17A (IL-17A) expression and circulating leucocyte subsets in hospitalised COVID-19 patients. Complete blood counts, C-reactive protein (CRP) levels, and lymphocyte subset profiles were measured, and cytokine concentrations were quantified by cytometric bead array.

**Results:**

Compared with controls, COVID-19 patients exhibited marked CD4^+^ lymphopenia and leucocytosis driven by neutrophilia and elevated immature granulocytes. Significantly higher expression of IFN-γ (*p* = 0.0153), IL-6 (*p* < 0.0001), IL-10 (*p* < 0.0001), IL-17A (*p* = 0.0310), and TNF-α (*p* = 0.0034) was observed in COVID-19 patients compared to uninfected participants. Cytokine-based stratification revealed that CRP was negatively associated with TNF-α (*p* = 0.0289) and positively associated with IL-10 (*p* = 0.0046) expression. Furthermore, TNF-α positivity correlated with altered distributions of eosinophils (*p* = 0.0217), monocytes (*p* = 0.0063), CD45^+^ lymphocytes (*p* = 0.0252), and CD19^+^ B cells (*p* = 0.0152). While, IFN-γ and IL-17A expression was linked to neutrophilia (*p* = 0.0439 and *p* = 0.0075 respectively). IL-10 positivity was associated with elevated immature granulocytes (*p* < 0.0001), CD16^+^CD56^+^ natural killer cells (*p* = 0.0116), and neutrophils (*p* = 0.0227). Following Benjamini–Hochberg correction for multiple comparisons, immature granulocytes remained the only immune cell subset with a significant distribution difference across IL-10–defined groups (*q* = 0.001). A Spearman’s rank-order correlation analysis was performed to assess the correlation between serum IL-6 levels and peripheral blood leucocyte counts, including platelet counts. Although basophil count showed a moderately positive correlation (*r_s_* = 0.4071, *p* = 0.0169) and IL-6 and platelet count showed a moderately negative connection (*r_s_* = −0.3770, *p* = 0.0234), these correlations were no longer significant following Benjamini–Hochberg correction for multiple comparisons.

**Conclusions:**

These findings reveal distinct cytokine-associated immunotypes marked by cell-specific shifts suggestive of emergency granulopoiesis and myelopoiesis. Our data provide an immunophenotypic framework for understanding COVID-19 pathogenesis and may inform targeted diagnostics and immunomodulatory strategies.

## 1 Introduction

Severe Acute Respiratory Syndrome Coronavirus 2 (SARS-CoV-2), a betacoronavirus, causes coronavirus disease 2019 (COVID-19), a respiratory disease that was considered a pandemic by the World Health Organisation in 2020 (1–3). Due to the rapid course of events, the COVID-19 pandemic placed unprecedented strain on healthcare systems globally (4, 5).

There is substantial diversity in the course of SARS-CoV-2 infection; at least 14% of infected patients experience severe symptoms, which are often linked to immune responses, dominated by local and systemic inflammation due to high levels of pro-inflammatory cytokines (6). Acute respiratory distress syndrome (ARDS) and cytokine storm syndrome have been observed in patients with severe COVID-19, and both conditions may ultimately result in mortality (7–9). The term “cytokine storm” describes an overabundance of pro-inflammatory cytokines that are excessively and repeatedly expressed, resulting in tissue injury, acute vascular leakage, and perhaps multi-organ failure (10, 11). It has been observed that dysregulated immune responses result in increased cytokine production, which may be crucial in immunopathogenesis (12). Determining the fundamental processes leading to disease heterogeneity has proven to be a challenging task. Thus far, it has been difficult to gather evidence that thoroughly explains the role and mode of action of the cytokines implicated in the cytokine storm, which include the production of IL-1 in macrophages, IL-1 stimulates the production of other pro-inflammatory cytokines such as tumour necrosis factor alpha (TNF-α), interferon gamma (IFN-γ), C-reactive proteins including interleukin 6 (IL-6), interleukin 10 (IL-10) and interleukin 17 (IL-17) (13–15). High levels of proinflammatory cytokines may lead to elevated nitric oxide and prostaglandins, which cause severe inflammation with respiratory distress and death. One approach that can help elaborate the disease heterogeneity of COVID-19 is through patient stratification and exploring the links between cytokines and effector immune cells.

Severe cases of COVID-19 are marked by elevated levels of pro-inflammatory cytokines (16–18). Although there has been recognition that elevated levels of cytokines correlate strongly with disease prognosis and severity, the relationship between these elevated cytokines and the distribution of peripheral immune cells is not yet fully comprehended. These relationships are particularly important especially in African cohorts where host genetic, environmental, and epidemiological factors may uniquely shape immune responses (19, 20). As a result, understanding cytokine-cell population relationships becomes clinically relevant in the stratification of patients according their biological immunotypes and revealing the mechanistic links between immune mediators and effector cell dynamics. Once these dynamics are well understood, novel biomarkers, treatments strategies and therapeutic targets can be postulated.

It is imperative to comprehend the cytokine storm events and immune cell dynamics reported in COVID-19 patients to design efficacious treatment strategies. Immune response patterns and COVID-19 progression exhibit a proximal link, similar to earlier research on SARS-CoV and MERS-CoV, and may be important factors in determining the severity of the illness (7, 21). The aim of this study was to investigate the association between TNF-α, IFN-γ, IL-10, IL-6 and IL-17A expression and the distribution of circulating leucocyte subsets in hospitalised COVID-19 patients. This analysis provides an immuno-phenotypic perspective on COVID-19, which may potentially inform specific diagnostics and therapeutics.

## 2 Materials and methods

### 2.1 Study participants and clinical data

The study received ethical approval from Zimbabwe’s Medical Research Council (MRCZ/A/2602). The study complied with the International Declaration of Helsinki, the Medical Research Council’s Ethical requirements for Research, and Zimbabwe’s Good Clinical Practice requirements. The study comprised 28 healthy participants who served as controls and 43 participants with COVID-19 infection who were recruited from Parirenyatwa General Hospital in Harare, Zimbabwe. Healthy control participants were tested and confirmed to lack detectable antibodies against SARS-CoV-2 using the lateral flow immunochromatographic assay. Participants were enrolled between July 1, 2020, and November 30, 2020, without regard for predetermined inclusion criteria other than a positive COVID-19 status. The reverse transcriptase polymerase chain reaction (RT-PCR), which probes for the presence of SARS-CoV-2 genetic material, was used to confirm that all recruited subjects had COVID-19 positive status as part of the hospital’s protocols. Demographic data was collected as part of hospital procedures, including the participants age and gender. Each newly enrolled patient had peripheral blood drawn during hospital admission and put in ethylenediaminetetraacetic acid (EDTA) vacutainers for further testing. Serum was also collected in storage vials and were immediately and stored at -80 °C until analysis to avoid repeated freeze–thaw cycles.

### 2.2 Determination of complete blood counts

A haematology analyser, Sysmex XN-3000™ (Sysmex Corporation, Kobe, Japan) was used to determine the enrolled patients’ complete blood counts. A volume of 88 μL of whole blood was aspirated in order to determine the counts within two hours of receipt. The differential white blood cell count (lymphocytes, neutrophils, eosinophils, and immature granulocytes) was determined using the white blood cell differential (WDF) channel. The global cell counts were determined using the white cell nucleated (WNR) channel. The haematology analyser used radio frequencies (RF), direct current (DC), cyanide-free sulfolyser technique, hydrodynamic focussing, and fluorescence flow cytometry to automatically determine the haematological parameters. The whole blood count values were captured and gathered for subsequent testing.

### 2.3 Flow cytometric determination of lymphocyte subsets

Lymphocyte subsets were quantified using the BD Multitest™ IMK kit with BD Trucount™ tubes (Becton, Dickinson and Company, BD Biosciences, San Jose, CA, USA) according to the manufacturer’s instructions. The 10× lysing solution supplied with the kit was diluted with deionised water to prepare a 1× working solution. For each sample of whole blood, two 12 × 75 mm BD Trucount™ tubes were labelled with the sample identification number and designated as tube A or B. The bead pellet at the bottom of each tube was inspected to confirm its integrity before use. Tube A was loaded with 20 μL of BD Multitest™ CD3/CD8/CD45/CD4 reagent [FITC-conjugated anti-CD3 (clone SK7), PE-conjugated anti-CD8 (clone SK1), PerCP-conjugated anti-CD45 (clone 2D1, HLe-1), APC-conjugated anti-CD4 (clone SK3)], and tube B with 20 μL of BD Multitest™ CD3/CD16+CD56/CD45/CD19 reagent [FITC-conjugated anti-CD3 (clone SK7), PE-conjugated anti-CD16 (clone B73) and anti-CD56 (clone NCAM16.2), PerCP-conjugated anti-CD45 (clone 2D1, HLe-1), APC-conjugated anti-CD19 (clone SJ25C1)]. A volume of 50 μL of well-mixed, anticoagulated whole blood was added to the bottom of each tube using reverse pipetting to ensure proper mixing with the reagents while avoiding disruption of the bead pellet. Tubes were capped, gently vortexed, and incubated for 15 minutes in the dark, at room temperature. Following incubation, 450 μL of the 1× lysing solution was added to each tube, followed by gentle vortexing and a further 15-minute incubation in the dark and at room temperature. Each sample was then acquired on a BD FACSCalibur™ flow cytometer (serial no. E97500097; BD Biosciences) and analysed using BD Multiset™ software (version 3.1 for Mac OS^®^, BD Biosciences), which automatically enumerated the lymphocyte subsets.

### 2.4 Qualitative analysis of serum C-reactive protein of COVID-19 patients

A qualitative examination of the serum C-reactive protein was conducted using a C-reactive protein latex agglutination test kit (Fortress Diagnostics Limited, Antrim, Northern Ireland, United Kingdom). A 50 μL serum sample and a positive control drop were placed in separate black circles on a card, which was supplied with the kit. After reconstituting the latex reagent, each black circle was filled with a drop of latex reagent. The latex reagent was uniformly dispersed within the circle to guarantee a thorough mixing of the mixture. The cards were then spun for two minutes at a speed of 100 revolutions per minute. Agglutination clumps that resembled the positive control (C-reactive protein of at least 6 mg/l) indicated a positive result. The procedure was repeated for all serum samples, and results were recorded for analysis.

### 2.5 Analysis of serum cytokine concentrations

Serum samples from COVID-19 patients were subjected to cytokine analysis using the BD™ Human Th1/Th2/Th17 cytometric bead array (CBA) kit (Becton, Dickinson and Company BD Biosciences, San Jose, California, United States of America) according to the manufacturer’s instructions. The kit enabled the simultaneous detection of TNF-α, IFN-γ, interleukin 10 (IL-10), interleukin 2 (IL-2), interleukin 4 (IL-4), interleukin 6 (IL-6), and interleukin 17A (IL-17A) and has theoretical limits of detection in the low pg/mL range for all analytes (~2.6 pg/mL for IL-2, ~4.9 pg/mL for IL-4, ~3.8 pg/mL for TNF-α, ~18.9 pg/mL for IL-17A, ~2.4pg/mL for IL-6, ~3.7pg/mL for IFN-γ, and ~4.5pg/mL for IL-10). Before the assay, the gating parameters of the instruments were set using the BD CellQuest Pro™ software (Becton, Dickinson and Company BD Biosciences, San Jose, California, United States of America). Collected serum samples were equilibrated at room temperature before use and 50 μL of each sample were aliquoted into separate tubes after equilibration. Lyophilized cytokine standards were reconstituted in 2 ml of assay diluent for a period of 15 minutes. Following the 15 minute equilibration at room temperature, the standards were serially diluted in two-fold dilution starting with a 1:2, and the final dilution was 1:256 in the assay diluent. After vigorously vortexing the capture beads cocktail, the mixture was centrifuged at 200 g using a Hermle ZK364 centrifuge (Maschinenfabrik Berthold Hermle AG, Gosheim, Germany). After centrifugation, the capture beads were aspirated and re-suspended in serum enhancement buffer, adding the volume lost during aspiration. A volume of 50 μL of mixed capture beads was added to all tubes containing 50 μL of aliquoted neat serum sample [COVID-19 hospitalised patients and healthy controls] and cytokine standards. To facilitate binding, the samples were left in a dark atmosphere for a whole night. Following incubation, the samples were washed with 1 ml of wash buffer and centrifuged at 200 g for 5 minutes. After centrifuging, the pellet was again suspended in 300 μL of wash buffer. All samples were acquired using the BD FACSCalibur™ flow cytometer (Becton, Dickinson and Company, BD Biosciences, San Jose, California, United States of America), and the data was recorded using an acquisition template. The FCAP Array™ application (version 3.0 for Windows^®^ OS Becton, Dickinson and Company BD Biosciences, San Jose, California, United States of America) was then used to import data for analysis. The software computed the mean fluorescence intensities (MFIs), which were fitted to a logistic curve-fitting equation to determine the concentrations of the cytokines. The determined cytokine concentrations were recorded for future investigation.

### 2.6 Data analysis

After all the results were captured, statistical analyses were done. All data were tested for normality prior to applying non-parametric tests. The Mann-Whitney *U* test was used to compare the cytokine expression and cellular populations of the participants and Fischer’s exact test was used to investigate association between c-reactive protein expression and cytokine expression. The correlation between serum IL-6 levels and peripheral blood leucocyte counts, including platelet counts, was evaluated using Spearman’s rank-order correlation analysis. Spearman’s correlation coefficient (*r_s_*) was used to estimate correlation strength and direction, and statistical significance was assessed with a two-tailed test. All data was analysed using statistical software, STATA (version 16.0, StataCorp Limited Liability Company, Texas, USA) and Graphpad Prism 9^®^ (Version 9.0, Graph pad Software Inc, San Diego, United States of America). Results with *p*-values less than 0.05 (< 0.05), were statistically significant. Comparisons were performed using Mann–Whitney *U* tests between cytokine-stratified groups. Raw *p*-values and Benjamini–Hochberg false discovery rate–adjusted *q*-values are shown. Statistical significance was defined as *q* < 0.05.

## 3 Results

### 3.1 Demographic characteristics of recruited COVID-19 patients

This study aimed to investigate the association between the expression of selected cytokines and various immune cells. Thus, 43 hospitalised COVID-19 participants were involved in this study; 13 (30.23%) of the participants were female, and 30 (69.77%) participants were male, according to a demographic study (**Table 1**). The median age was 49 years, with an interquartile range of 40 to 60 years.

**Table 1:** Demographic characteristics of the patients recruited for the study.

Variable	Hospitalised COVID-19 participants	Control group
N	43	28
Age in years	48.72 (40, 60)	26.52 (21, 33)
Sex:		
Female, *n* (%)	13 (30.23)	17 (60.71)
Male, *n* (%)	30 (69.77)	11 (39.29)

### 3.2 Summary of haematological features and lymphocyte profiles

COVID-19 infected participants had normal total white blood cells (WBCs), red blood cell (RBCs) counts and Haemoglobin levels. However, the percentage of lymphocytes were lower than the normal range (**Tables 2**, **3**). These participants had high levels of CD8^+^CD4^-^ T cells and CD19^+^ B lymphocytes, however the frequency of CD4^+^CD8^-^ T lymphocytes and natural killer cells (CD56^+^ CD16^+^) were lower than the normal range. Consequently, CD4^+^CD8^-^ T-cell lymphopenia was identified as a characteristic of COVID-19 patients. Additionally, there was a bias toward lower lymphocyte percentages and greater neutrophil and immature granulocyte percentages.

**Table 2:** Summary of haematological characteristics of hospitalised COVID-19 patients.

Variable	Hospitalised COVID-19 patients	Reference range
White Blood Cell Count (x 10^9^/L)	10.045 (7.665, 12.585)	4.5 – 11
Red Blood Cell Count (x 10^12^/L)	4.43 (3.385, 4.93)	4.65 – 6.5
Haemoglobin Count (g/dl)	12.46 (10.25, 14.15)	13 – 18
Haematocrit (%)	43 (33.75, 47.7)	43 – 55
Mean Corpuscular Volume (fl)	93.95 (88.6, 103.7)	77 – 95
Mean Corpuscular Haemoglobin (pg)	28.55 (27.15, 30.2)	27 – 32
Mean Corpuscular Haemoglobin Concentration (g/dl)	29.5 (28.25, 31.3)	32 – 36
Red Cell Distribution Width Coefficient (%)	16.25 (14.45, 17.7)	11.5 – 14.5
Red Cell Distribution Width Standard Deviation (fl)	56.63545 (49, 60.6)	40 – 55
Platelets (x 10^9^/L)	206.5 (149.5, 336)	140 – 440
Neutrophil Count (x 10^9^/L)	7.145 (3.93, 10.75)	2 – 7.5
Neutrophil Percentage (%)	78.55 (60.55, 85.55)	51 – 76
Lymphocyte Count (x 10^9^/L)	1.105 (0.69, 2.085)	1 – 4
Lymphocyte Percentage (%)	13.1 (6.5, 23.75)	20 – 40
Eosinophil Count (x 10^9^/L)	0.01 (0, 0.03)	0 – 0.45
Eosinophil Percentage (%)	0.1 (0, 0.4)	0 – 5
Monocyte Count (x 10^9^/L)	0.58 (0.17, 0.85)	0.18 – 0.8
Monocyte Percentage (%)	5.3 (2.35, 8.4)	5 – 8
Basophil Count (x 10^9^/L)	0.025 (0.01, 0.04)	0 – 0.2
Basophil Percentage (%)	0.3 (0.13, 0.6)	0 – 0.2
Immature Granulocyte Count (x 10^9^/L)	0.31 (0.1, 0.62)	0 – 0.03
Immature Granulocyte Percentage (%)	2.7 (1.2, 6.4)	0 – 0.5
Neutrophil to Lymphocyte Ratio	6.02(2.5, 12.2)	–

**Table 3:** Summary of lymphocyte profiles of hospitalised COVID-19 patients.

Variable	COVID-19 patients median (Q1, Q3)	Reference range
CD3^+^ Lymphocyte Percentage (%)	51 (37, 70)	55 – 84
CD3^+^ Lymphocyte Count (cells/μL)	666 (269, 1340)	690 – 2540
CD8^+^ CD4^-^ Lymphocyte Percentage (%)	27 (17, 35)	13 – 41
CD8^+^ CD4^-^ Lymphocyte Count (cells/μL)	273 (144, 571)	190 – 1140
CD4^+^ CD8^-^ Lymphocyte Percentage (%)	12 (2, 28.98)	31 – 60
CD4^+^ CD8^-^ Lymphocyte Count (cells/μL)	132 (29, 388)	410 – 1590
CD16^+^/CD56^+^/CD16^+^CD56^+^ Lymphocyte Percentage (%)	5 (3, 15)	5 – 27
CD16^+^/CD56^+^/CD16^+^CD56^+^ Lymphocyte Count (cells/μL)	79.5 (42, 206)	90 – 590
CD19^+^ Lymphocyte Percentage (%)	17.5 (6, 32)	6 – 25
CD19^+^ Lymphocyte Count (cells/μL)	326 (63, 572)	90 – 660

### 3.3 Overview of the expression of cytokines in COVID-19 patients

**Figure 1:**
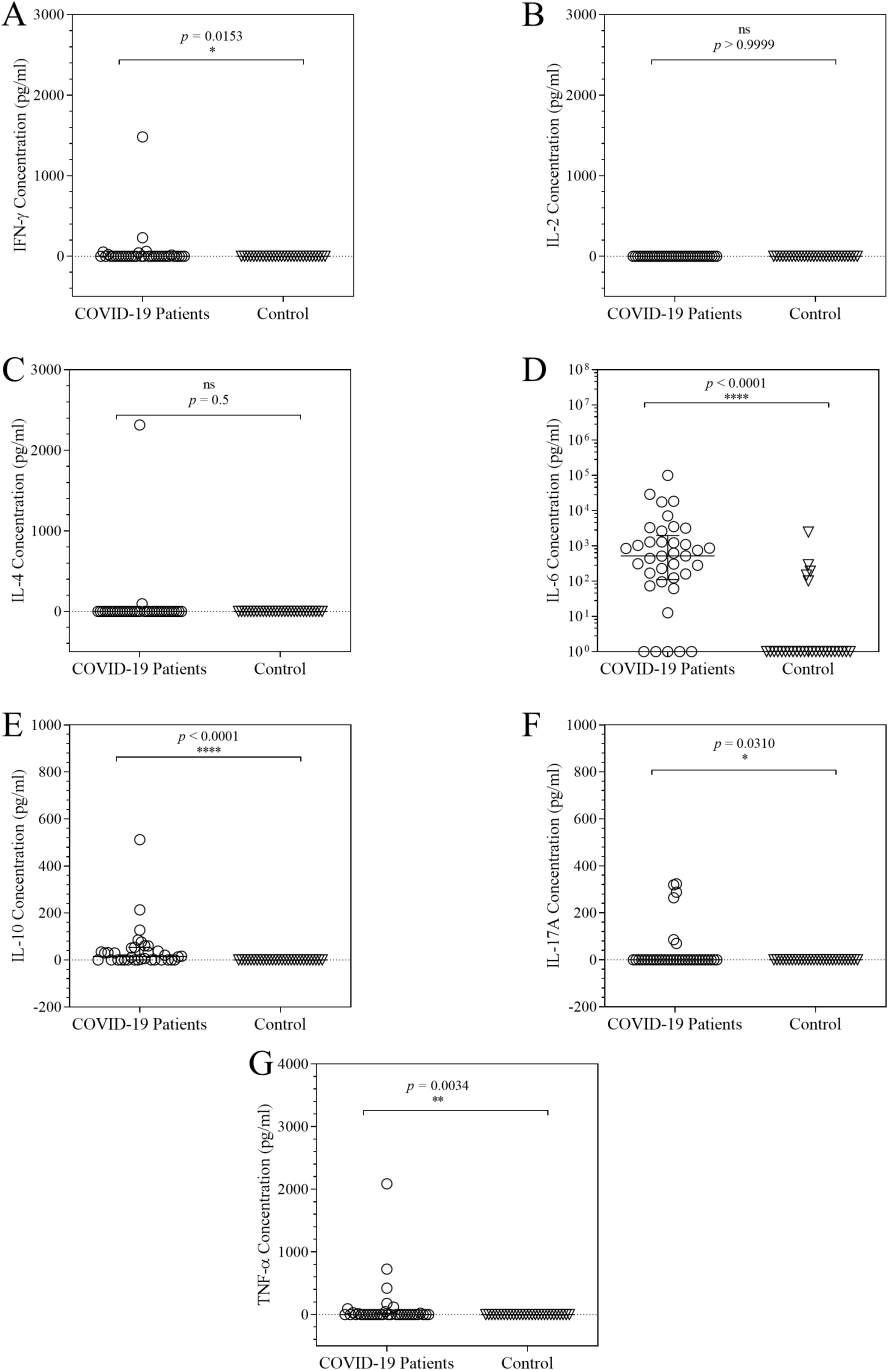
Column scatter plots of COVID-19 patients’ cytokine expression. On every graph, the cytokine concentration is shown. The cytokine concentrations of each patient are shown in a scatter plot with the controls, displaying the median and interquartile ranges to summarize the distribution of the cytokines, IFN-γ **(A)**, IL-2 **(B)**, IL-4 **(C)**, IL-6 **(D)**, IL-10 **(E)**, IL-17A **(F)** and TNF-α **(G)**. To analyse the data, the distributions were compared using the Mann-Whitney U test at a 95% significant interval. The asterisks (*) indicate statistical difference in distribution between a pair of groups, where * = *p* < 0.05; ** = *p* < 0.01; **** = *p* < 0.0001. ns = no significant statistical difference.

A Th1/Th2/Th17 cytometric bead array was utilized to compare serum cytokine concentrations between hospitalised COVID-19 patients and healthy uninfected participants. IFN-γ, IL-6, IL-10, IL-17A, and TNF-α, were considerably expressed in COVID-19 patients as compared to the uninfected participants (**Figure 1**). However, IL-2 and IL-4 serum cytokine concentrations, were noticeably low. The highest statistically significant correlation with COVID-19 was seen in the cytokines IL-6 and IL-10 (*p* < 0.0001; *p* < 0.0001). Strong statistically significant associations were also observed for TNF-α (*p* = 0.003), IFN-γ (*p* = 0.0153), and IL-17A (*p* = 0.0310).

### 3.4 Association between C-reactive protein and cytokine expression

Serum samples were used for the qualitative investigation of C-reactive protein (CRP) expression. The Fischer’s exact test was then used to analyse the data and ascertain if TNF-α expression and CRP expression were related. Upon analysis of the data, a negative association was observed between the expression of CRP and TNF-α (*p* = 0.0289). However, a strong positive association between CRP and IL-10 expression was also observed (*p* = 0.0046) (**Table 4**). Whilst TNF-α and IL-10 exhibited significant associations, IFN-γ expression status (*p* = 0.5585) and IL-17A expression status (*p* = 0.5585) exhibited no significant association to CRP status.

**Table 4:** Distribution of C-reactive protein with respect to cytokine expression.

Laboratory Test	COVID-19 patients		*p*
	C-reactive protein negative	C-reactive protein positive	
TNF-α:			
Positive, *n* (%)	4 (80.00)	7 (24.14)	0.0289
Negative, *n* (%)	1 (0.00)	22 (75.86)	
IFN-γ:			
Positive, *n* (%)	0 (0.00)	6 (20.69)	0.5585
Negative, *n* (%)	5 (100.00)	23 (79.31)	
IL-10:			
Positive, *n* (%)	0 (0.00)	21 (72.41)	0.0046
Negative, *n* (%)	5 (100.00)	8 (27.59)	
IL-17A:			
Positive, *n* (%)	6 (20.69)	0 (0.00)	0.5585
Negative, *n* (%)	23 (79.31)	5 (100.0)	

### 3.5 Distribution of cellular counts in COVID-19 patients stratified by TNF-α, IFNγ, IL-10 and IL-17A expression

**Figure 2:**
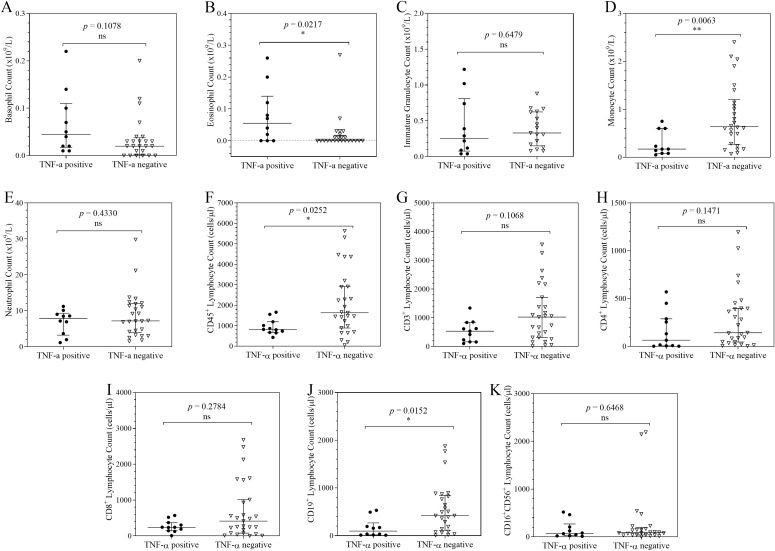
Column scatter plots of cell counts in COVID-19 patients stratified by TNF-α expression. Each graph shows the plots of the cell counts of each patient. The patients were categorised according their TNF-α expression status and the median and interquartile ranges of the 2 categories summarise the distribution of the leucocytes: Basophils **(A)**; Eosinophils **(B)**; Immature Granulocytes **(C)**; Monocytes **(D)**; Neutrophils **(E)**; CD45+ Lymphocytes **(F)**; CD3+ Lymphocytes **(G)**; CD4+ Lymphocytes **(H)**; CD8+ Lymphocytes **(I)**; CD19+ Lymphocytes **(J)**; CD16+CD56+ Lymphocytes **(K)**. To analyse the data, the distributions were compared using the Mann-Whitney *U* test at a 95% significant interval. The asterisks (*) indicate statistical difference in distribution between a pair of groups, where * = *p* < 0.05; ** = *p* < 0.01. ns = no significant statistical difference.

**Figure 3:**
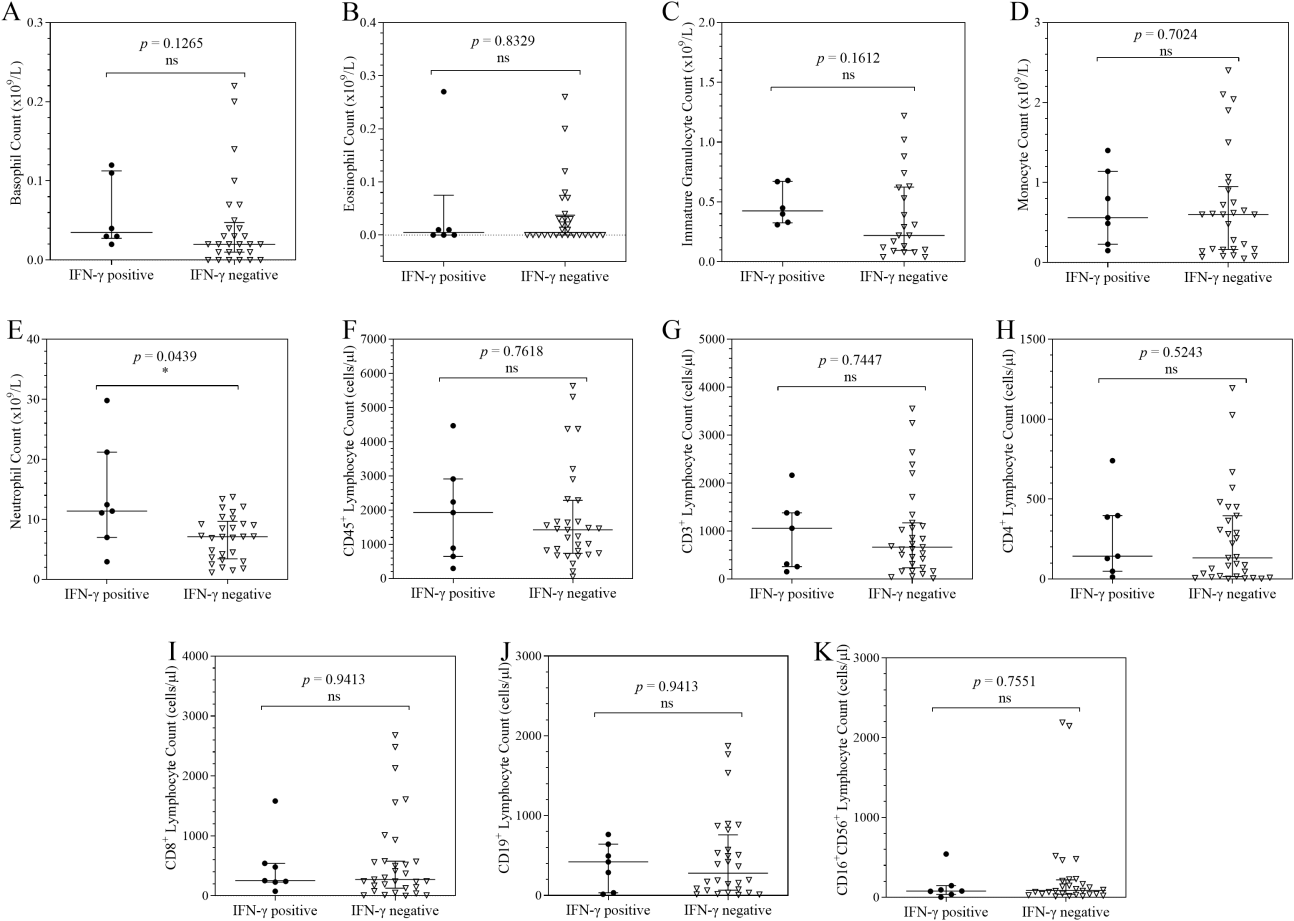
Column scatter plots of cell counts in COVID-19 patients stratified by IFN-γ expression. Each graph shows the plots of the cell counts of each patient. The patients were categorised according their IFN-γ expression status and the median and interquartile ranges of the 2 categories summarise the distribution of the leucocytes: Basophils **(A)**; Eosinophils **(B)**; Immature Granulocytes **(C)**; Monocytes **(D)**; Neutrophils **(E)**; CD45^+^ Lymphocytes **(F)**; CD3^+^ Lymphocytes **(G)**; CD4^+^ Lymphocytes **(H)**; CD8^+^ Lymphocytes **(I)**; CD19^+^ Lymphocytes **(J)**; CD16^+^CD56^+^ Lymphocytes **(K)**. To analyse the data, the distributions were compared using the Mann-Whitney U test at a 95% significant interval. The asterisks (*) indicate statistical difference in distribution between a pair of groups, where * = *p* < 0.05. ns = no significant statistical difference.

**Figure 4:**
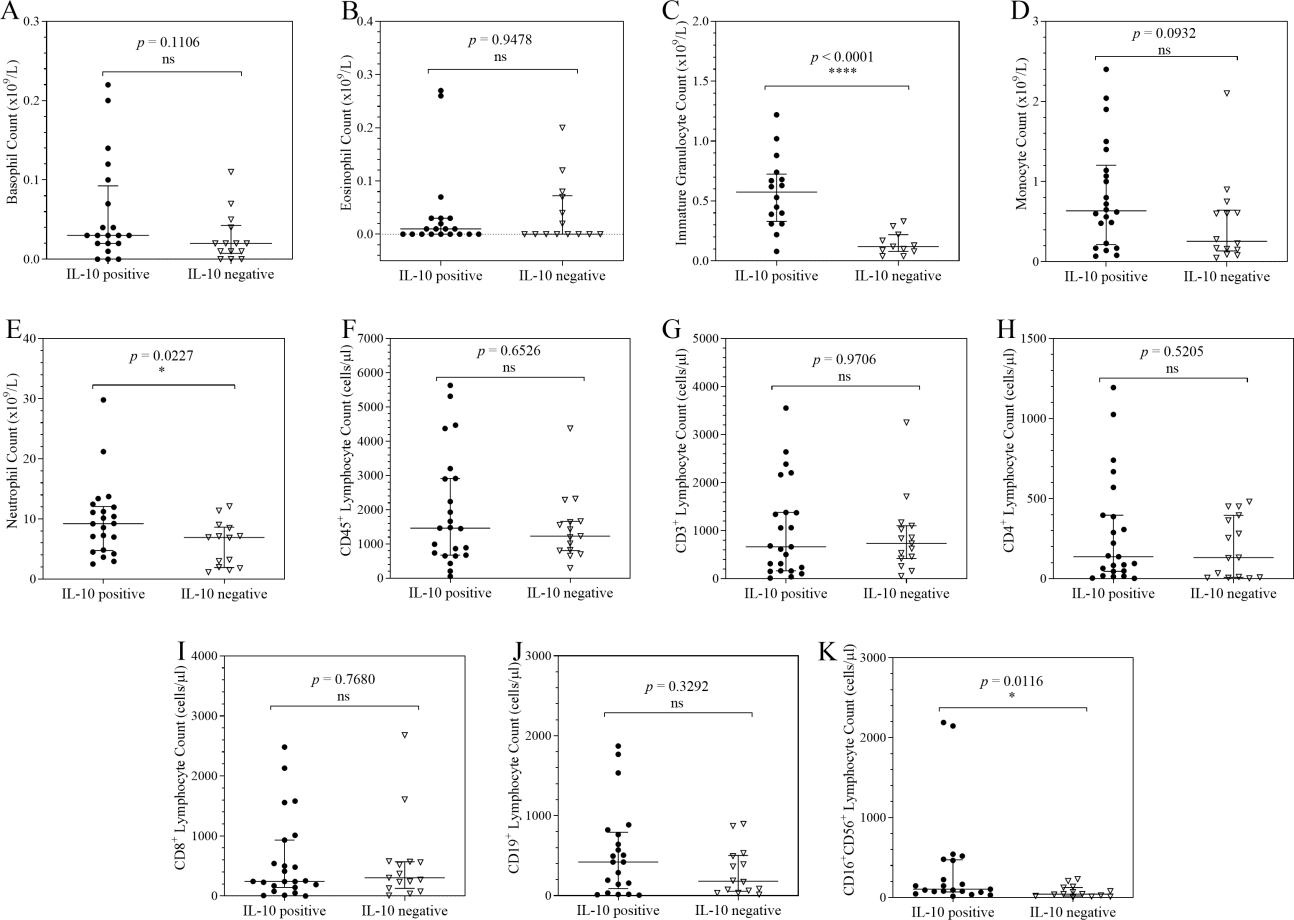
Column scatter plots of cell counts in COVID-19 patients stratified by IL-10 expression. Each graph shows the plots of the cell counts of each patient. The patients were categorised according their IL-10 expression status and the median and interquartile ranges of the 2 categories summarise the distribution of the leucocytes: Basophils **(A)**; Eosinophils **(B)**; Immature Granulocytes **(C)**; Monocytes **(D)**; Neutrophils **(E)**; CD45^+^ Lymphocytes **(F)**; CD3^+^ Lymphocytes **(G)**; CD4^+^ Lymphocytes **(H)**; CD8^+^ Lymphocytes **(I)**; CD19^+^ Lymphocytes **(J)**; CD16^+^CD56^+^ Lymphocytes **(K)**. To analyse the data, the distributions were compared using the Mann-Whitney U test at a 95% significant interval. The asterisks (*) indicate statistical difference in distribution between a pair of groups, where * = *p* < 0.05. ns = no significant statistical difference.

The distribution of cellular counts stratified by TNF-α, IFN-γ, IL-10 and IL-17A expression was analysed for each leucocyte subset to distinguish different immune response phenotypes in COVID-19 (**Figures 2**–**4**). When compared to the TNF-α negative patients, TNF-α positive patients had significantly higher eosinophil counts (*p* = 0.0217), and significantly lower monocyte (*p* = 0.0063), CD45^+^ lymphocyte (*p* = 0.0252), and CD19^+^ lymphocyte counts (*p* = 0.0152). Basophil, immature granulocyte, neutrophils, CD3^+^ lymphocyte, CD4^+^ lymphocyte, CD8^+^ lymphocyte and CD16^+^CD56^+^ lymphocyte counts showed no statistically significant difference in distribution between the 2 groups.

Furthermore, patients who were IFN-γ positive had greater neutrophil counts than those who were IFN-γ negative (*p* = 0.0439), making neutrophils the only leucocyte subgroup to show a statistically significant difference in distribution.

When compared to the IL-10 negative subgroup, the IL-10 positive patient subgroup was shown to have significantly higher counts of neutrophils, immature granulocytes, and CD16^+^CD56^+^ lymphocytes (**Figure 4**). The results showed a very strong and significant statistical difference in the distribution of immature granulocytes in which IL-10 positive participants had higher of immature granulocyte counts compared to their negative counterparts (*p* < 0.0001). There was also a significant statistical difference in the distribution of CD16^+^CD56^+^ lymphocyte counts (*p* = 0.0116) and neutrophil counts (*p* = 0.0227). There were no statistically significant differences in the distributions of basophil, eosinophil, monocyte, CD45^+^ lymphocyte, CD3^+^ lymphocyte, CD4^+^ lymphocyte, CD8^+^ lymphocyte, and CD19^+^ lymphocyte counts.

**Figure 5:**
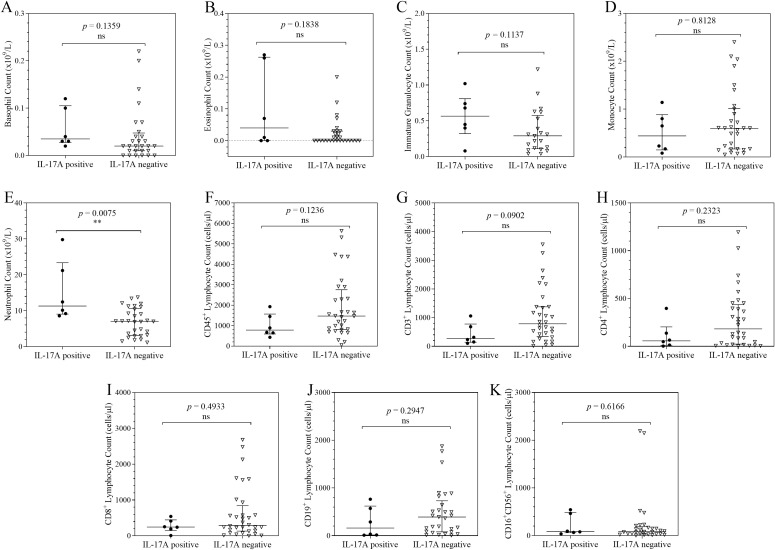
Column scatter plots of cell counts in COVID-19 patients stratified by IL-17A expression. Each graph shows the plots of the cell counts of each patient. The patients were categorised according their IL-17A expression status and the median and interquartile ranges of the 2 categories summarise the distribution of the leucocytes: Basophils **(A)**; Eosinophils **(B)**; Immature Granulocytes **(C)**; Monocytes **(D)**; Neutrophils **(E)**; CD45^+^ Lymphocytes **(F)**; CD3^+^ Lymphocytes **(G)**; CD4^+^ Lymphocytes **(H)**; CD8^+^ Lymphocytes **(I)**; CD19^+^ Lymphocytes **(J)**; CD16^+^CD56^+^ Lymphocytes **(K)**. To analyse the data, the distributions were compared using the Mann-Whitney U test at a 95% significant interval. The asterisks (*) indicate statistical difference in distribution between a pair of groups, where ** = *p* < 0.01. ns = no significant statistical difference.

When the neutrophil counts of IL-17A positive and IL-17A negative individuals were examined, there was a statistically significant difference (*p* = 0.0075) in their distribution (**Figure 5**). Patients who tested positive for interleukin 17A had noticeably higher neutrophil populations than those who tested negative. While neutrophils had a significantly different distribution, basophils, eosinophils, immature granulocytes, monocytes, CD45^+^ lymphocytes, CD3^+^ lymphocytes, CD4^+^ lymphocytes, CD8^+^ lymphocytes, CD19^+^ lymphocytes, and CD16^+^CD56^+^ lymphocytes did not.

After significant associations were revealed for immune cell distributions across cytokine-stratified, the unadjusted *p*-values were adjusted using the Benjamini–Hochberg false discovery rate (**Table 5**). Statistical significance was defined at *q* < 0.05 for the false discovery rate correction. Only immature granulocytes differed significantly between IL-10–defined groups (*q* = 0.0011). All the other trends for all sets of results were not significant after false discovery rate correction.

**Table 5:** Raw *p*-values and Benjamini–Hochberg false discovery rate–adjusted *q*-values for immune cell subsets across cytokine-defined patient groups

	TNF-α Distribution		INF-γ Distribution		IL-10 Distribution		IL-17A Distribution	
	*p*-value	*q*-value	*p*-value	*q*-value	*p*-value	*q*-value	*p*-value	*q*-value
Basophils	0.1078	0.1976	0.1265	0.5911	0.1104	0.2429	0.1359	0.2990
Eosinophils	0.0217	0.0693	0.8329	0.9894	0.9478	0.9706	0.1838	0.3370
Immature Granulocytes	0.6479	0.6479	0.1612	0.5911	0.0001	0.0011	0.1137	0.2990
Monocytes	0.0063	0.0693	0.7024	0.9894	0.0932	0.2429	0.8128	0.8128
Neutrophils	0.4330	0.5292	0.0439	0.4829	0.0227	0.0832	0.0075	0.0825
CD45^+^ Lymphocytes	0.0252	0.0693	0.7618	0.9894	0.6526	0.8973	0.1236	0.2990
CD3^+^ Lymphocytes	0.1068	0.1976	0.7447	0.9894	0.9706	0.9706	0.0902	0.2990
CD4^+^ Lymphocytes	0.1471	0.2312	0.5243	0.9894	0.5205	0.8179	0.2323	0.3650
CD8^+^ Lymphocytes	0.2784	0.3828	0.9413	0.9894	0.7680	0.9387	0.4933	0.6029
CD19^+^ Lymphocytes	0.0152	0.0693	0.9894	0.9894	0.3292	0.6035	0.2947	0.4052
CD16^+^CD56^+^ Lymphocytes	0.6468	0.6479	0.7551	0.9894	0.0116	0.0638	0.6166	0.6783

### 3.6 Correlation between IL-6 levels and circulating leucocyte and platelet counts

The correlation between IL-6 levels and haematological parameters was assessed using Spearman’s rank correlation analysis. Basophil count showed a moderately positive correlation (*r_s_* = 0.4071, *p* = 0.0169), whereas IL-6 and platelet count showed a moderately negative connection (*r_s_* = −0.3770, *p* = 0.0234) (**Table 6**). However, these correlations were no longer statistically significant for platelet count (*q* = 0.1404) and basophil count (*q* = 0.1404) following false discovery rate (FDR) correction using the Benjamini–Hochberg approach. Monocyte, immature granulocyte, neutrophil, eosinophil, CD45^+^ lymphocyte, CD3^+^ T cell, CD4^+^ T cell, CD8^+^ T cell, NK cell, and CD19^+^ B cell counts did not show any statistically significant correlations with IL-6.

**Table 6:** Spearman correlation analysis between IL-6 levels and hematologic parameters.

Parameter	Spearman *r* (*r_s_*)	*p*-value	*q*-value
Platelets (×10^9^/L)	-0.3770	0.0234	0.1404
Neutrophil Count (×10^9^/L)	0.2101	0.2188	0.4376
Eosinophil Count (×10^9^/L)	-0.0669	0.7068	0.7711
Monocyte Count (×10^9^/L)	-0.1400	0.4153	0.5537
Basophil Count (×10^9^/L)	0.4071	0.0169	0.1404
Immature Granulocyte Count (×10^9^/L)	0.1180	0.5578	0.6694
CD45^+^ Lymphocytes (cells/µL)	-0.2277	0.1693	0.5158
CD3^+^ Lymphocytes (cells/µL)	-0.1723	0.3009	0.4376
CD8^+^CD4^-^ Lymphocytes (cells/µL)	-0.2523	0.1265	0.4376
CD4^+^CD8^-^ Lymphocytes (cells/µL)	0.2045	0.2180	0.5199
CD16^+^CD56^+^ NK Cells (cells/µL)	-0.1640	0.3466	0.7776
CD19^+^ B Lymphocytes (cells/µL)	0.0495	0.7776	0.4376

## 4 Discussion

Impaired immune function seems to be a major factor in the most severe COVID-19 patients. Specifically, increased levels of IL-2, IL-7, granulocyte colony-stimulating factor (G-CSF), IFN-γ, interferon gamma-induced protein 10 (IP-10) and TNF-α are associated with ARDS and organ failure, which are characteristic of a severe disease course (10, 22). These traits are similar to ARDS brought on by cytokine release syndrome (CRS) and linked to infections with SARS-CoV and MERS-CoV (7). Similar to previous research on SARS-CoV and MERS-CoV, immune response patterns and COVID-19 development demonstrate a proximal relationship and may be crucial factors in explaining the pathology of the disease (7, 21). There could be a proximal relationship between levels of circulating cytokines and peripheral immune cell, which could help reveal the dominant immunotypes or even map the course of severe COVID-19. Consequently, the goal of this study was to determine how TNF-α, IFN-γ, IL-10 and IL-17 may be implicated in divergent cytokine-associated immunotypes. Discovering these connections might help develop therapeutic strategies as well as comprehend the underlying mechanisms and how immunological abnormalities are mapped in severe COVID-19 cases.

Among the 43 COVID-19 patients analysed, 69.77% of the patients were male. The median age and interquartile range of these patients were 48.72 (40, 60) years. The study’s demographic findings were consistent with those of a prior retrospective analysis (23). Analysis of the patients’ full blood counts revealed that the percentages of leucocyte subsets were skewed towards higher proportions of neutrophils and immature granulocytes. Although the proportions of immature granulocytes and neutrophils were higher than those of other subsets, the populations and percentages of CD4^+^ T-lymphocytes were lower in most individuals. Similarly, meta-analysis study found that while lymphocyte numbers decline, neutrophil counts rise (24). Consequently, populations of neutrophils, immature granulocytes, and CD4^+^ T-lymphocytes may have a major impact on the immunopathology of COVID-19. Increased coagulation, NETosis, and prolonged innate immune responses are the outcomes of such an imbalance, and they may promote the formation of cytotoxic granules at infection sites (25).

The cytokine levels in COVID-19 patients were significantly higher than those in the control group for IFN-γ, IL-6, IL-10, IL-17A, and TNF-α, whereas the cytokine levels in the blood for IL-2 and IL-4 were significantly lower. It should be noted that the theoretical limits of detection for all analytes are: ~2.6 pg/mL for IL-2; ~4.9 pg/mL for IL-4; ~3.8 pg/mL for TNF-α; ~18.9 pg/mL for IL-17A; ~2.4pg/mL for IL-6; ~3.7pg/mL for IFN-γ; and ~4.5pg/mL for IL-10). Previous investigations had shown that severe cases of COVID-19 were characterised by a cytokine storm, which was regarded as indicative of a macrophage activation syndrome (10; 1, 26). This work stands out in part because it characterised immune responses in a sub-Saharan African cohort, which is a group that is under-represented in COVID-19 immunological research. According to our results, there is an indication of dysregulated immune responses, systemic inflammation. In order to uncover or validate the claims of macrophage activation syndrome, we sought to examine the potential roles of TNF-α, IFN-γ, IL-10, and IL-17A in distinct cytokine-associated immunotypes. As such, our findings reveal discrete leucocyte distribution patterns associated with TNF-α, IFN-γ, IL-10 and IL-17A expression in COVID-19 patients, underscoring the functional heterogeneity of cytokine-driven immune responses.

Patients were stratified based on their cytokine expression profiles, and associations with CRP status were examined. Both TNF-α and IL-10 showed significant correlations with CRP. The TNF-α and CRP relationship aligns with the physiological inflammatory cascade, suggesting that TNF-α is expressed early in the course of infection. In contrast, the IL-10 and CRP association likely reflects a counter-regulatory response, in which IL-10 is produced to modulate high levels of systemic inflammation. Although these results provide significant insights, a more robust method may have been employed using longitudinal sampling and quantitative analysis. While our cross-sectional design limits direct temporal inference, findings from longitudinal studies consistently demonstrate a similar pattern, with TNF-α peaking early and IL-10 rising as a subsequent anti-inflammatory response (27, 28).

This study aimed to explore cytokine-driven immune cell shifts that may occur during the course of severe COVID-19. TNF-α positivity was associated with depletion of peripheral monocytes, CD19^+^ lymphocytes, and CD45^+^ lymphocytes. This pattern may reflect TNF-α mediated apoptosis and possibly tissue trafficking during acute infection. Such a phenotype is clinically relevant, as high TNF-α on admission together with lymphopenia has been associated in other studies with poor prognosis, including increased risk of ICU admission and death (10, 28, 29). IL-10 positivity was associated with marked increases in neutrophils, immature granulocytes, and natural killer lymphocytes. This IL-10 positive immunophenotype may represent a paradoxical compensatory state in which IL-10 acts to dampen hyperinflammation, yet coincides with emergency granulopoiesis, emergency myelopoiesis, and natural killer lymphocyte activation. Notably, despite strong anti-inflammatory signalling, this phenotype did not coincide with recovery of lymphocyte populations, suggesting that regulatory responses may fail to restore adaptive immunity in the context of sustained inflammation. Experimental evidence supports the mechanistic plausibility of these observations, in which *in vivo* models have demonstrated that IL-10 can trigger emergency myelopoiesis by inducing an IFN-γ dependent transcriptional programme in hematopoietic progenitors (30) and can promote myeloid output by protecting progenitors from apoptosis (31). While these mechanisms are supported by experimental systems, our cross-sectional data cannot establish causality, and the extent to which these processes occur in human COVID-19 remains to be fully elucidated. Finally, IFN-γ and IL-17A positivity were both associated with neutrophil mobilisation. Our previous work demonstrated a significant correlation between IL-17A expression and an elevated neutrophil-to-lymphocyte ratio (NLR), implicating the T-helper 17 axis in COVID-19 immunopathogenesis (32). Taken together, IFN-γ positive and IL-17A positive immunophenotypes may define a neutrophil dominant inflammatory state that could further suppress adaptive immune responses. This cohort’s cytokine and leucocyte patterns are generally in line with findings from Asian, European, and North American studies showing that hospitalised COVID-19 patients have changed leucocyte distributions and elevated pro-inflammatory cytokines (10, 28, 33, 34). These findings imply that, in spite of population-specific diversity, fundamental inflammatory processes remain conserved globally.

Although various immune-cell subsets had nominal associations with cytokine-defined stratification, adjustment for multiple testing revealed that only immature granulocyte distribution remained statistically significant, particularly within IL-10-defined groups. The results show that the majority of the identified associations did not pass multiple-testing correction, indicating that there are very limited significant associations between cytokine stratification and immune cell distributions in this sample. Immature granulocyte imbalances continue after adjustment, which is noteworthy given that the growth of immature myeloid populations has been associated to inflammatory stress and emergency myelopoiesis in acute infection (30, 31). However, given the cross-sectional methodology and minimal correction for clinical factors, these results should be regarded with caution and viewed as hypothesis-generating rather than mechanistic evidence of cytokine-driven immune remodelling.

Interleukin-6 is a classical pro-inflammatory cytokine secreted by various cell types that regulates haematopoiesis, induces acute-phase proteins, and modulates T-lymphocyte responses, including the lymphocyte dysfunction seen in SARS-CoV infection (35, 36). Given its wide range of immunomodulatory effects, we used Spearman’s rank correlation analysis to determine the relationship between circulating immune cell subsets and IL-6 concentration. IL-6 extensively expressed among the hospitalised COVID-19 patients, limiting effective categorisation by cytokine expression; hence, a correlation-based approach was adopted. Initial analysis suggested moderate associations between IL-6 concentration and both basophil and platelet counts; however, these correlations did not remain statistically significant following false discovery rate (FDR) adjustment. The observed inverse relationship between IL-6 and platelet count may reflect inflammation-associated platelet consumption and potential thrombotic activation, consistent with the recognised role of IL-6 in promoting systemic inflammatory and procoagulant responses (37). Nonetheless, the limited sample size may have reduced statistical power to detect subtle but biologically relevant associations. Larger cohorts are warranted to clarify whether these trends represent true biological relationships.

According to our data, COVID-19 patients significantly expressed IFN-γ, IL-6, IL-10, IL-17A, and TNF-α. When the patients were stratified based on the expression of IFN-γ, IL-10, IL-17A, and TNF-α, important cell shifts and immunological patterns were revealed. While TNF-α appeared to be expressed during the course of infection that may be involved in TNF-α mediated apoptosis and tissue trafficking during the acute, IL-10, IL-17A and IFN-γ appeared to be involved in hyperinflammatory events that are neutrophilic and IL-10 may implicated in emergency granulopoiesis and emergency myelopoiesis. This study’s cross-sectional design precludes temporal or causal inference, therefore, conclusions are difficult to draw in this case. Although there was considerable homogeneity with regards to the ethnicity of recruited participants, the data was not collected and is a limitation of this study. Sample size was modest and limited to a single center. Another limitation of this study is that data on co-morbid infections common in sub-Saharan Africa, such as malaria, helminth infections, and HIV, were not systematically collected. As a result, we were unable to assess their potential confounding effects on immune responses and COVID-19 outcomes in this population. Longitudinal studies with functional assays are needed to validate these findings, highlighting the aspects on medication use, symptom onset and viral loads, which are important with respect to COVID-19, which were not presented in this study. While these associations reveal important immune cell shifts, our data set was cross-sectional and could not establishment important clues that predict causality.

## Data Availability

The raw data supporting the conclusion of this article will be made available by the authors, without undue reservation.
